# Separating the Empirical Wheat From the Pseudoscientific Chaff: A Critical Review of the Literature Surrounding Glyphosate, Dysbiosis and Wheat-Sensitivity

**DOI:** 10.3389/fmicb.2020.556729

**Published:** 2020-09-25

**Authors:** Jacqueline A. Barnett, Deanna L. Gibson

**Affiliations:** ^1^Department of Biology, The University of British Columbia, Kelowna, BC, Canada; ^2^Department of Medicine, Faculty of Medicine, The University of British Columbia, Kelowna, BC, Canada

**Keywords:** gut microbiome, dysbiosis, glyphosate, Roundup, crop-desiccation, mental health, wheat intolerance, non-celiac gluten sensitivity

## Abstract

The prevalence of digestive disorders has increased globally, as countries have adopted a more “Westernized” diet pattern. A Western diet, characterized as high in fat and refined carbohydrates, can also be defined as a product of increased technology and industrialization. Modern farmers rely on agrochemicals to meet the needs of a growing population, and these chemicals have shifted the Western diet’s chemical composition. While the number of individuals choosing to live a wheat-free lifestyle without a celiac disease diagnosis has increased, clinical trials have shown that gluten from wheat is not responsible for causing symptoms in healthy individuals suggesting that something else is inducing symptoms. The herbicide, glyphosate, is applied to wheat crops before harvest to encourage ripening resulting in higher glyphosate residues in commercial wheat products within North America. Glyphosate inhibits the shikimate pathway, a pathway exclusive to plants and bacteria. Glyphosate’s effect on dysbiosis was not considered when making safety recommendations. Here, we evaluate the literature surrounding glyphosate’s effects on the gut microbiome and conclude that glyphosate residues on food could cause dysbiosis, given that opportunistic pathogens are more resistant to glyphosate compared to commensal bacteria. However, research on glyphosate’s effects on the microbiome suffers from numerous methodological weaknesses, and these limitations make it impossible to draw any definitive conclusions regarding glyphosate’s influence on health through alterations in the gut microbiome. In this review, we critically evaluate the evidence currently known and discuss recommendations for future studies.

## Digestive Diseases and Crop Desiccation

**FIGURE 1:**
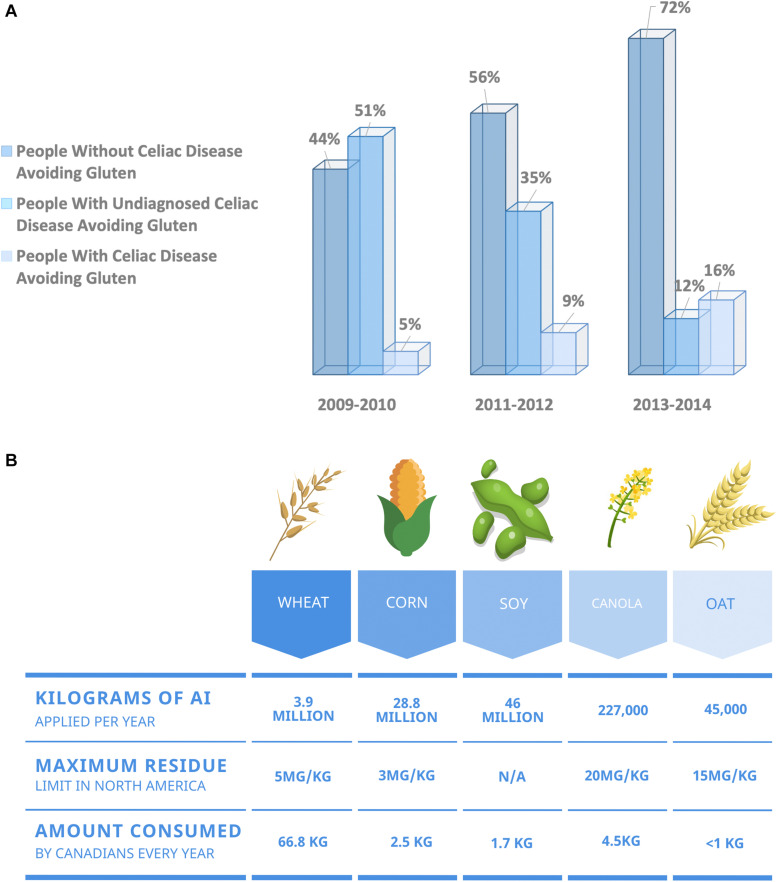
**(A)** Over the past decade, more North Americans are choosing to exclude wheat from their diet, citing health benefits in the absence of a celiac disease diagnosis. North America has seen a growing increase in the number of individuals adopting a gluten-free diet in the absence of a celiac disease diagnosis. Data obtained from the National Health and Nutrition Examination Survey. Data presented as percentage of respondents (Choung et al., 2017). **(B)** Summary of maximum residue limits allowed for glyphosate on the five most consumed food crops in North America (Health Canada, 2015).

Digestive disorders cost North Americans an estimated $154 billion annually in healthcare costs and lost productivity (Fedorak et al., 2012; Peery et al., 2019). Canada has the highest incidence of digestive diseases in the world, with two-thirds of Canadians suffering from a gastrointestinal condition within a given year (Fedorak et al., 2012). Some of these disorders are chronic inflammatory conditions, including inflammatory bowel disease and celiac disease. However, many digestive disorders plaguing North Americans are non-specific, eluding diagnosis based on any one set of criteria. Over the past decade, North America has seen a growing increase in the number of individuals choosing to live a wheat-free lifestyle in the absence of a celiac disease diagnosis (Figure 1A). When surveyed, individuals who abstain from wheat-based foods report experiencing less gastrointestinal discomfort and improved digestive health, reduced inflammation, reduced joint pain, and improved mental health (Health Canada, 2014). Often, individuals attribute the act of going *gluten-*free to their improved health and wellbeing (Niland and Cash, 2018). However, double-blind, randomized clinical trials have implicitly shown that gluten from wheat is not responsible for symptoms in non-celiac and otherwise healthy individuals (Croall et al., 2019). Is it possible that agricultural practices we have embraced in the past two decades are responsible for this dramatic increase in wheat-sensitivity?

One agricultural practice that gained popularity during the 1990s is the desiccation of crops, including wheat. Desiccation refers to the process of applying a chemical to a plant before harvest to kill vegetation. Desiccation corrects for uneven growth and is common in regions where the growing season is short and damp. Cereal grains, including wheat, are particularly prone to uneven ripening resulting in an increased prevalence of desiccation (Figure 1B). Glyphosate is a systemic desiccant with broad-spectrum herbicidal action taking weeks to dry crops. However, glyphosate has the added benefit of controlling green weeds and therefore is one of the most commonly used commercial desiccants.

Some European countries, including Italy, have banned the use of glyphosate pre-harvest while others, including France and Germany, plan to ban its use entirely by 2023. North America is one of the most prolific glyphosate users, with over 25 million kilograms purchased annually in Canada (Health Canada, 2012), and over 36 million kilograms applied annually in the United States (Benbrook, 2016). Routine monitoring of 3,188 food-items collected by the Canadian Food Inspection Agency (CFIA) found that 29.7% of items surveyed contained glyphosate residues (Canadian Food Inspection Agency, 2017). Data collected by the CFIA also revealed that pre-harvest application of glyphosate on wheat crops is leading to higher glyphosate residues within the Canadian food supply. Of the 3,188 samples tested, 869 were grain products. In total, 36.6% of the grain-based products tested contained glyphosate residues, and 3.9% contained residues over the maximum limit currently set for cereal crops (Canadian Food Inspection Agency, 2017). To understand the possible implications of these findings and how glyphosate might influence human health, one must first understand its underlying mechanism of action.

## Glyphosate Targets Types of Bacteria Present in the Gut Microbiome

**FIGURE 2:**
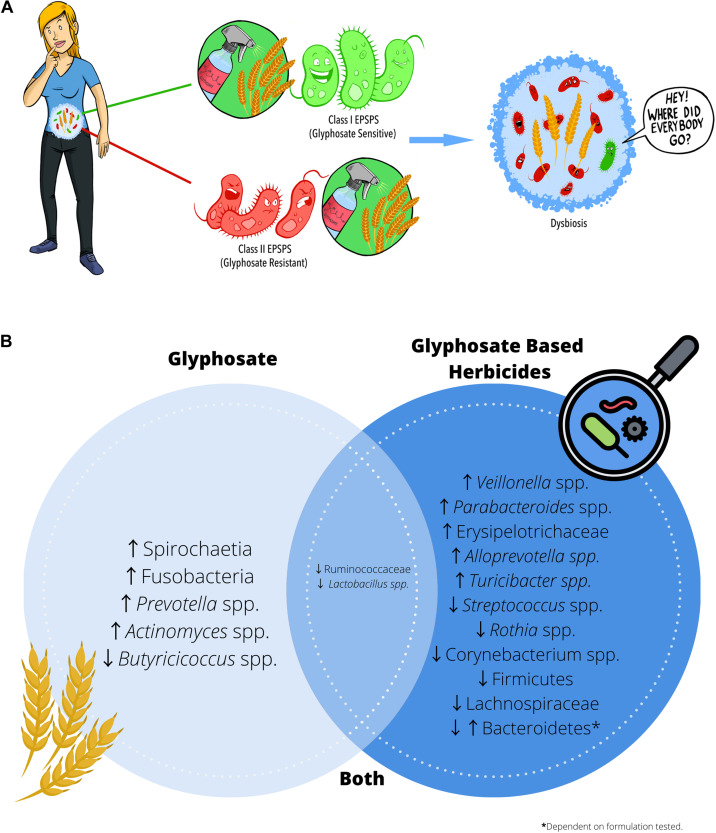
Glyphosate residues present on food may cause intestinal dysbiosis. **(A)** Glyphosate exhibits its herbicidal action through inhibition of the shikimate pathway enzyme EPSPS. Class I EPSPS are sensitive to the effects of glyphosate and are found in all plants and bacteria. However, glyphosate resistant EPSPS (Class II) appear to be more prevalent in opportunistic pathogens and may contribute to dysbiosis. **(B)** Summary of the alterations in microbial composition reported in the literature when administering either glyphosate or glyphosate-based herbicides.

Glyphosate exhibits its herbicidal action through inhibition of the shikimate pathway, a seven-step metabolic pathway where carbon skeletons from carbohydrate metabolism are converted to chorismate. Glyphosate acts as a competitive inhibitor of the enzyme 5-enolpyruvylshikimate-3-phosphate synthase (EPSPS), preventing the synthesis of chorismate. Chorismate is vital for many plant functions, including aromatic amino acid, hormone, and vitamin synthesis. Mammals do not possess the shikimate pathway or any of the enzymes, which is why glyphosate was considered to be non-toxic to humans. However, recent studies have highlighted the potential cytotoxic and carcinogenic effects of glyphosate both *in vivo* and *in vitro* (Van Bruggen et al., 2018). In addition to direct toxicity, it is possible that glyphosate could influence health through secondary means via the gut microbiome, which harbors trillions of microorganisms living as a functional ecosystem. The shikimate pathway is essential for bacterial survival that some organisms have developed glyphosate resistance. Class I EPSPS enzymes are found within all plants and bacteria and are highly sensitive to the effects of glyphosate (Molin, 1998). Class II enzymes have been characterized in a subset of bacteria and are more common in pathogenic species, including *Staphylococcus aureus* and *Streptococcus pneumonia* (Sutton et al., 2016). In the absence of Class II enzymes, some bacteria, including *Escherichia coli*, have developed mutations that mitigate the harmful effects of glyphosate (Cao et al., 2012) and these advantageous mutations appear to be more common in pathogenic isolates (Bote et al., 2019). Commensal bacteria appear to be more susceptible to glyphosate, as they are more likely to possess glyphosate-sensitive Class I EPSPS enzymes than potentially pathogenic bacteria, thereby promoting dysbiosis (Figure 2A). Literature often describes gut dysbiosis as an overabundance of opportunistic pathogens, including *E. coli* and *S. aureus*, and this imbalance is associated with increased inflammation (Sannasiddappa et al., 2011; Kittana et al., 2018), obesity (Gao et al., 2015) and altered behavior (Jang et al., 2018). In essence, symptoms that individuals report a reduction in when eliminating wheat from their diet.

When determining glyphosate’s toxicity, the highest level that does not produce harmful effects is referred to as the no-observed-effect-level (NOEL) (Reyna, 1985). The acceptable daily intake (ADI) is the amount of glyphosate that can be ingested daily without discernible health risk; (Reyna, 1985) and is determined by dividing the NOEL by a safety factor (commonly 100) (Renwick, 1993). However, different governing bodies may err on the side of caution and use a higher safety factor, leading to an array of ADI values globally. For instance, the Environmental Protection Agency (EPA), the acting executive agency of the United States, has the highest ADI for glyphosate globally, currently set at 1.75 mg/kg body weight/day (Mao et al., 2018). For comparison, the ADI established by Europe and Canada is 0.5 mg and 0.3 mg/kg body weight/day (Nielsen et al., 2018), respectively. However, only direct glyphosate toxicity was considered when determining the NOEL. Alarmingly, glyphosate’s influence over health through secondary means, such as the gut microbiome, was never considered. Given that the gut microbiome is critical for our overall health and disease susceptibility, glyphosate residues on wheat may contribute to dysbiosis, thereby affecting our overall health. To understand the secondary effects of glyphosate on human health through dysbiosis, we reviewed the literature and critically evaluated the evidence surrounding glyphosate’s effects on the gut microbiome. Given the magnitude of the EPA dose compared to other countries, any study using a dose higher than the EPA’s ADI will be referred to as “high-dose.”

## Glyphosate Exposure Induces Gut Dysbiosis

All bacteria contain glyphosate-sensitive Class I EPSPS enzymes; however, the degree to which bacteria succumb to its effect differs considerably. Opportunistic pathogens in the gut, are more likely to contain Class II EPSPS enzymes that are resistant to glyphosate. Studies using high-dose glyphosate exposure drives dysbiosis increasing opportunistic pathogens, including members of the phyla Fusobacteria (Tang et al., 2020). The phylum Fusobacteria contains both commensal organisms and pathogenic species; however, an increased abundance of Fusobacteria has been associated with the development of colorectal cancer (Park et al., 2016). High-dose glyphosate exposure has also been correlated with increases in other bacterial groups, including *Prevotella* spp. and *Actinomyces* spp. (Mao et al., 2018). An increased abundance of rod-shaped bacteria including, *Prevotella* spp. (Lerner et al., 2017), and *Actinomyces* spp. (Ou et al., 2009) is a potential risk factor in celiac disease development in susceptible individuals. These findings suggest that high-dose glyphosate exposure may promote opportunistic pathogen expansion in the gut microbiome.

The extinction of commensal bacteria also contributes to gut dysbiosis. Indeed, animal studies examining the impact of glyphosate on the microbiome at doses ranging from 5 mg–500 mg/kg body weight/day have shown that glyphosate decreases bacterial species commonly hypothesized to be beneficial, including *Lactobacillus* spp. (Mao et al., 2018) and *Butyricicoccus* spp. (Dechartres et al., 2019). *Lactobacillus* spp. constitutes a significant component of the human microbiota in several sites throughout the digestive tract, including stomach, duodenum and jejunum (Walter, 2008). *Lactobacillus* spp. tend to exhibit a mutualistic relationship with humans by protecting against pathogenic infections in exchange for nutrients from their human host. As its name implies, members of the genus *Butyricicoccus* spp. are significant producers of the short-chain fatty acid butyrate, which is essential in the maintenance of gastrointestinal health through inhibition of pro-inflammatory pathways and the reduction of oxidative stress within the colon (Canani et al., 2011). Butyrate is also the primary energy source of colonic epithelial cells, and adequate levels aid in maintaining barrier function (Konig et al., 2016). A failure to maintain barrier function homeostasis has been implicated in chronic inflammation (Canani et al., 2011; Konig et al., 2016), and high-dose glyphosate exposure causes higher levels of pro-inflammatory cytokines including IL-1β, IL-6 and TNF-α in addition to increased transcription of mitogen-activated protein (MAP) kinase and nuclear factor kappa-light-chain-enhancer of activated B cells (NF-kB) within the small intestine (Mao et al., 2018). In addition to increasing inflammation and oxidative stress, reduced butyrate levels influence intestinal motility (Walter, 2008), which has been associated with a host of digestive symptoms including abdominal pain, diarrhea and reflux (Martinucci et al., 2014). While these studies suggest that glyphosate alone may induce dysbiosis, in practice, crops are sprayed with glyphosate-based herbicides (GBH), which contain many additives in addition to glyphosate. These additives, alone or combined with glyphosate, could have differential effects on bacterial communities present within the gut.

## Commercial Herbicide Adjuvants Further Drive Dysbiosis

The literature has shown that some bacterial communities that are resistant to glyphosate exposure are less able to withstand commercial herbicide exposure. Indeed, glyphosate and GBH share some similarities, like decreases in *Lactobacillus spp*. (Mao et al., 2018; Tang et al., 2020). However, the differences in microbial composition observed with GBH exposure are dependent on the formulation tested. For example, exposure to the herbicide R Grand Travaux Plus^®^ results in a significant increase in the Bacteroidetes phyla (Lozano et al., 2018). Whereas exposure to the glyphosate-based herbicide Roundup^®^ resulted in a significant *decrease* in Bacteroidetes (Aitbali et al., 2018). Bacteroidetes are considered one of the most stable phyla of the gastrointestinal bacteriome, and they serve a broad range of metabolic functions for their host. A reduction in Bacteroidetes has been shown to be associated with obesity (Koliada et al., 2017), whereas an overabundance has been associated with irritable bowel syndrome (Pittayanon et al., 2019). There were other notable differences observed between commercial formulations. Exposure to Roundup 3Plus^®^ resulted in significant decreases in Lachnospiraceae and increased Erysipelotrichaceae (Dechartres et al., 2019). Lachnospiraceae has been shown to be protective against colon cancer in humans through its production of butyric acid (Meehan and Beiko, 2014). In contrast, the increased abundance of Erysipelotrichaceae has been implicated in the development of colon cancer (Kaakoush, 2015). These findings suggest that herbicide adjuvants may induce alterations to the gut microbiome and may have a synergistic effect when used in combination with glyphosate. However, it is essential to note that the range of doses examined varied considerably, with some studies using relatively small amounts [50 ng/L (Lozano et al., 2018)] to enormous doses [500 mg/kg body weight (Tang et al., 2020)] to elicit a response. An interesting theme in the literature is that the deleterious effects of both glyphosate and GBH do not appear to be dose-dependent. To truly understand the potential implications of glyphosate exposure on the gut microbiome and human health, it is vital to examine doses that have been previously deemed safe for human exposure.

## Pre and Post-Natal Glyphosate and Glyphosate-Based Herbicide Exposure May Influence Early Microbiome Development

Early-life exposure to EPA approved levels of glyphosate or GBH, results in significant changes to the developing neonatal microbiome in a mouse model (Mao et al., 2018). While pregnant dams exposed to glyphosate or the herbicide Roundup^®^ did not display dysbiosis, pups exposed during gestation and throughout weaning showed altered gut microbiome diversity, including reductions in *Lactobacillus* and an increase in Bacteroidetes (*Prevotella* spp.) (Mao et al., 2018). Pups exposed to Roundup^®^ also had alterations to other communities, including a reduction in *Streptococcus* spp., and *Rothia* spp., and increases in *Veillonella* spp., as well as *Parabacteroides* spp., (Mao et al., 2018) again highlighting the possible additive effects of GBH formulations. Given the neonatal gut microbiome plays such a critical role in immune development and tolerance (Mazmanian and Round, 2009) the dysbiosis caused by glyphosate could have catastrophic consequences for immunity. Indeed, subspecies of the *Rothia* genus have been identified as playing a critical role in the degradation of gluten within the mouth and upper gastrointestinal tract (Zamakhchari et al., 2011). Gluten proteins are difficult to digest by mammalian proteolytic enzymes and recent studies have highlighted microorganism derived enzymes which aid in breaking down these proteins (Zamakhchari et al., 2011). *Rothia* spp., contain not only the enzymes necessary for protein degradation but also have enzymes that target the immunogenic epitopes that play a crucial role in celiac disease (Zamakhchari et al., 2011). These findings suggest that exposure to glyphosate, either alone or in a commercial preparation, at doses previously deemed safe for human health, may have profound effects on microbiome development and may be an environmental trigger in the development of celiac disease.

## Glyphosate and Glyphosate- Based Herbicide Exposure May Alter Behavior Through Changes in the Gut Microbiome

While there are many consequences to glyphosate-induced dysbiosis, one of the more pressing effects may be on our mental health. Recent studies show that dysbiosis can affect the gut-brain axis (Carabotti et al., 2015) a bidirectional communication system between the central nervous system and the gastrointestinal tract (Martinucci et al., 2014). Exposure to Roundup 3Plus^®^ during pregnancy significantly increased the abundance of *Turicibacter* spp., (Dechartres et al., 2019) which, in combination with Clostridiaceae, plays a critical role in the modulation of gut-derived serotonin (Reigstad et al., 2015). Serotonin is a monoamine neurotransmitter that elicits effects locally within the gastrointestinal tract regulating intestinal movements and secretion (Reigstad et al., 2015). Serotonin is also a key neurotransmitter in the gut-brain-microbiome axis (O’Mahony et al., 2015) and the intricate crosstalk between the gut microbiome and altered serotonergic neurotransmission have implications for mood and behavior (Fung et al., 2019). Indeed, pregnant dams exposed to either glyphosate alone or the herbicide Roundup 3Plus^®^, displayed altered licking behavior toward their pups and abnormal brain pathology (Dechartres et al., 2019). Exposure to Roundup^®^ is associated with increased anxiety and depression-like behaviors in mice, correlated with decreases in *Corynebacterium* spp., Firmicutes (*Lactobacillus* spp.) and Bacteroidetes (Aitbali et al., 2018). Research focused on the gut-brain-microbiome axis is in its infancy, and much remains unknown in this rapidly developing field. However, given that mood disorders are often comorbidities associated with digestive diseases, understanding the implications ubiquitous environmental toxins, including glyphosate, may have on the gut microbiome and behavior is of vital importance.

## Sheafing It Together

Over the past two decades, there has been a dramatic increase in the number of individuals reporting beneficial health effects when eliminating wheat from their diets. Exposure to glyphosate alone or through the administration of herbicide appears to promote gut dysbiosis through a reduction in commensal bacteria species, including *Lactobacillus* spp., (Aitbali et al., 2018; Mao et al., 2018; Tang et al., 2020) and butyrate-producing bacteria (Dechartres et al., 2019) and an increase in opportunistic pathogens (Mao et al., 2018; Dechartres et al., 2019; Tang et al., 2020). This imbalance may be due to the presence of glyphosate-resistant class II EPSPS enzymes which appear to be more common in opportunistic pathogens. However, the sequence of class II EPSPS enzymes appear to be unique to the particular strain they are isolated from (i.e., CP4 EPSPS, Ab EPSPS) and many Class II enzymes remain uncharacterized. The dysbiosis induced by glyphosate appears to favor several disease phenotypes including inflammation, (Kittana et al., 2018; Tang et al., 2020) reflux-disease, (Martinucci et al., 2014) obesity (Koliada et al., 2017) and colon cancer, (Park et al., 2016) and may be an important environmental trigger in the etiology of celiac disease through alterations in gluten-neutralizing bacteria (Mao et al., 2018) or the over-abundance of rod-shaped bacteria (Ou et al., 2009; Lerner et al., 2017). The effects of glyphosate on the gut microbiome can have systemic consequences through modulation of the serotonergic system which may have implications for behavior and could play a role in the development of mood disorders including anxiety and depression (Aitbali et al., 2018). Glyphosate may also have ramifications for early microbiome development when exposed both pre and postnatally (Mao et al., 2018).

While the current review focused on the agricultural practice of desiccating wheat, it should be noted that many crops, including legumes, corn, and soy, have been shown to contain high glyphosate residues due to desiccation and the advancement of glyphosate-resistant crops. Eliminating wheat from one’s diet does not guarantee the elimination of glyphosate exposure. However, wheat products have been shown in independent testing to contain higher residues post-processing (Canadian Food Inspection Agency, 2017) and make up a significant portion of the average North American’s dietary glyphosate exposure. Future studies examining other popular diet patterns, including gluten-free, ketogenic, paleo and the Mediterranean diet pattern, may offer unique insight with regards to dietary glyphosate exposure.

Research surrounding glyphosate’s effect on the gut microbiome has yielded conflicting results, with studies suggesting glyphosate has a limited impact on the gut microbiome (Nielsen et al., 2018) and others claiming it has extensive, detrimental effects (Aitbali et al., 2018; Lozano et al., 2018; Mao et al., 2018; Dechartres et al., 2019; Tang et al., 2020). How can there be so much variation in the data? Research examining glyphosate’s impact on gut health has primarily suffered from two major methodological flaws. First, dose matters – too much of anything, whether it be water or aspirin – has the potential to be detrimental to one’s health. Research surrounding glyphosate’s effects on gut health often use exaggeratedly high doses compared to what the average North American is exposed to through diet. Some studies promote the ADI as a physiologically relevant dose, however most ADI’s for glyphosate are much higher than what the average individual is exposed to through diet alone. Future studies examining dietary levels of glyphosate exposure on the gut microbiome are warranted to determine the actual risk of glyphosate induced dysbiosis. The second weakness has to do with the formulation. While glyphosate is the active ingredient, food crops are desiccated with GBH, which contain compounds in addition to glyphosate. Complicating matters further is the fact that most GBH are proprietary and their ingredients and the relative percentages are unknown. This ambiguity poses a significant challenge for researchers as they do not know what they’re working with, the amount present and the synergistic effects of these chemicals when combined. Additionally, crops are often treated with a proverbial “cocktail” of agrochemicals, including other herbicides, in addition to glyphosate and GBH. The cytotoxic effects of glyphosate appear to increase when combined with other herbicides, including Paraquat (Gunatilake et al., 2019). This synergistic phenomenon suggests that relatively low glyphosate residues within our food supply could have serious consequences when combined with other commonly used agrochemicals. Moreover, this synergistic phenomenon has never been studied on the composition of the gut microbiome.

Arguably, the best way to determine the effect of desiccated crops on the microbiome would be to examine the effects of consuming commercially available desiccated and non-desiccated crops on the microbiome composition. The duration of the experimental intervention may also have profound implications for microbial diversity. The studies included in the current review had exposure durations ranging from 2 weeks (Nielsen et al., 2018) to 2 years (Lozano et al., 2018) with the former reporting no significant alterations to the composition of the microbiome, even at doses of 25 mg/kg body weight/day (Nielsen et al., 2018). Often, the first foods introduced to infants are wheat and grain-based and, in the absence of intolerance, we continue eating wheat-based foods for the duration of our lives, meaning chronic glyphosate exposure throughout life. Studies have even suggested that certain taxa present within the gut microbiome are heritable (Goodrich et al., 2014; Beaumont et al., 2016) signifying that the effects of glyphosate exposure could be inherited and even compounded over time. Long term, multi-generational animal studies utilizing appropriate dietary exposure levels are necessary for determining the *actual* implications for human health. Furthermore, studies conducted examining the effects of glyphosate on the gut microbiome have only explored healthy populations. In many conditions, including inflammatory bowel disease and celiac disease, there is a combination of environmental and biological factors that culminate in the etiology of disease. Additionally, all studies included in this review are rodent studies. While mice are an invaluable tool in microbiome research, there are some dissimilarities in the composition of the gastrointestinal tract and mouse microbiome compared to that of a human. Future correlative studies examining the microbial composition of pesticide workers or individuals consuming a predominantly organic diet may shed light on the actual risk posed to humans. However, given how ubiquitous glyphosate is within the North American landscape, it would likely be impossible to find a true glyphosate-free control for comparison.

## Conclusion

Glyphosate exposure, either through active ingredient alone or commercial herbicide formulations, has the potential to induce dysbiosis by creating an imbalance between commensal members of the gastrointestinal microbiome and opportunistic pathogens. Glyphosate may be a critical environmental trigger in the etiology of several disease states associated with dysbiosis, including celiac disease, inflammatory bowel disease and irritable bowel syndrome. Glyphosate exposure may also have consequences for mental health, including anxiety and depression, through alterations in the gut microbiome. However, the research surrounding glyphosate’s effects on the gut microbiome also suffers from numerous methodological weaknesses including artificially high-doses, insufficient duration, proprietary ingredients and an over reliance on animal models. Future long-term studies examining physiologically relevant doses in both healthy and genetically susceptible populations are warranted to determine the real risk posed to human health.

## Author Contributions

JB critically reviewed and summarized all literature, drafted the figures and the manuscript. DG conceived of the study with insights from JB, provided oversight, supervised the project, and critically evaluated the manuscript. JB and DG edited the manuscript and approved the final version. Both authors contributed to the article and approved the submitted version.

## Conflict of Interest

The authors declare that the research was conducted in the absence of any commercial or financial relationships that could be construed as a potential conflict of interest.
